# Acute Vitamin D_3_ Supplementation in Severe Obesity: Evaluation of Multimeric Adiponectin

**DOI:** 10.3390/nu9050459

**Published:** 2017-05-05

**Authors:** Stefania Mai, Gillian E. Walker, Roberta Vietti, Stefania Cattaldo, Chiara Mele, Lorenzo Priano, Alessandro Mauro, Gianni Bona, Gianluca Aimaretti, Massimo Scacchi, Paolo Marzullo

**Affiliations:** 1 Laboratory of Metabolic Research, Ospedale S. Giuseppe, I.R.C.S.S. Istituto Auxologico Italiano, 28921 Piancavallo-Verbania, Italy; r.vietti@auxologico.it; 2 Laboratory of Paediatrics, Department of Health Sciences, Università del Piemonte Orientale, 28100 Novara, Italy; gillian.walker@med.uniupo.it (G.E.W.); gianni.bona@med.uniupo.it (G.B.); 3 Laboratory of Clinical Neurobiology, I.R.C.C.S. Istituto Auxologico Italiano, Ospedale S. Giuseppe, 28921 Piancavallo-Verbania, Italy; s.cattaldo@auxologico.it (S.C.); lorenzo.priano@unito.it (L.P.); alessandro.mauro@unito.it (A.M.); 4 Division of General Medicine, I.R.C.C.S. Istituto Auxologico Italiano, Ospedale S. Giuseppe, 28921 Piancavallo-Verbania, Italy; chiara.mele1989@gmail.com (C.M.); massimo.scacchi@unimi.it (M.S.); paolo.marzullo@med.uniupo.it (P.M.); 5 Endocrinology, Department of Translational Medicine, Università del Piemonte Orientale, 28100 Novara, Italy; gianluca.aimaretti@med.uniupo.it; 6 Department of Neuroscience, Università di Torino, 10124 Torino, Italy; 7 Department of Clinical Sciences and Community Health, Università di Milano, 20122 Milano, Italy

**Keywords:** multimeric adiponectin, vitamin D, obesity, weight loss

## Abstract

Obesity predisposes to vitamin D deficiency (VDD) and glucose abnormalities. It is currently debated if vitamin D administration may improve glucose homeostasis by interacting with modulators of insulin sensitivity, such as adiponectin and its oligomers. In a 4-week inpatient study on a metabolic rehabilitation program, consisting of individualized caloric restriction and aerobic physical exercise in obese subjects with VDD, we assessed the acute effects of 600,000 IU cholecalciferol given per os VD group, 12 subjects; body mass index (BMI) 42.7 ± 1.3 kg/m^2^) or placebo per os (PL group, 12 subjects, BMI 39.8 ± 0.9 kg/m^2^) on high (HWM-A), medium (MMW-A), and low molecular weight adiponectin (LMW-A), as quantified by western immunoblot (WIB) and ELISA. During the 4-week study, dieting promoted a similar magnitude of weight loss in VD and PL groups. Compared to the PL group, cholecalciferol administration increased 25(OH)Vit D levels (*p* < 0.001) and promoted a significant increase of HMW-A expression analyzed by WIB (*p* = 0.02). In parallel, a significant decrease of leptin/HMW-A ratio (*p* < 0.05), a biomarker of metabolic homeostasis, was observed. During the study, changes of MMW-A and LMW-A occurred independently of cholecalciferol administration, and were likely explained by weight loss. At odds with these findings, the ELISA assessment of adiponectin oligomers showed no modifications in the VD group or PL group. Current findings suggest that acute cholecalciferol administration selectively modifies HMW-A and the leptin/HMW-A ratio.

## 1. Introduction

Vitamin D is a fat-soluble secosteroid hormone that has an established physiological role in mineral and bone homeostasis. Its precursor vitamin D_3_, or cholecalciferol, is synthesized by 7-dehydrocholesterol in the skin during sun exposure, and it is hydroxylated first in the liver to form 25-hydroxycholecalciferol (calcidiol, or 25(OH)Vit D), and then in the kidney to generate the bioactive form 1,25(OH)_2_ Vit D, the actions of which are mediated by the vitamin D nuclear receptor (VDR). Notably, the VDR is widely expressed in human tissues, where it likely mediates the pleiotropic functions of vitamin D [1]. It has long been shown that serum 25(OH)Vit D concentrations are inversely correlated with body mass index (BMI), as well as with several anthropometric or biochemical surrogates of adiposity [2]. Vitamin D deficiency (VDD) affects up to 95% of patients with severe obesity [3], and an associative network relates VDD and obesity to low grade inflammatory state, insulin resistance, metabolic syndrome, and type 2 diabetes mellitus (T2DM) [3,4,5]. Evidence collected in case-control and observational studies led to the hypothesis that cholecalciferol administration could improve glucose tolerance, pancreatic beta-cell function, insulin and C-peptide responses, as well as insulin sensitivity [6,7,8]. However, the possibility that vitamin D_3_ supplementation may help to prevent the risk of T2DM has been challenged, and the ability of vitamin D_3_ supplementation to improve fasting glucose, HbA_1c_ levels, or insulin resistance in patients with impaired glucose tolerance (IGT), gestational diabetes, or T2DM is openly debated [9].

Being a source of adipokines, white adipose tissue (WAT) is actively involved in regulating many endocrine and metabolic functions related to energy storage, glucose homeostasis, and cardiometabolic health [10]. In particular, adiponectin is a WAT-derived adipokine that acts as an insulin-sensitizer and is capable of yielding anti-inflammatory and anti-atherogenic effects [11]. Because the secretion of adiponectin is negatively regulated by adiposity, particularly visceral fat accumulation [12], adiponectin levels are reduced in overweight/obese and insulin resistant states [13], and increase upon bodyweight reduction [14]. It has long been shown that adiponectin circulates as three isoforms with largely different molecular weights. The high molecular weight adiponectin isoform (HMW-A) is the recognized major determinant of insulin-sensitizing effects of adiponectin in liver, muscle, and endothelial tissue, as compared to medium (MMW-A) and low molecular weight adiponectin (LMW-A) [15,16]. In previous investigations, serum HMW-A expression was positively associated with indices of insulin sensitivity and high density lipoprotein (HDL) cholesterol, while being negatively associated with BMI, central body fat, hyperinsulinemia, and T2DM [17,18,19]. In obesity, HMW-A levels are significantly decreased but increase during weight loss [20,21]. Cross-sectional studies found a positive association between circulating adiponectin and 25(OH)Vit D levels [22,23,24], as well as between VDD and hypoadiponectinemia [25,26]. While meta-analytic data failed to prove stimulatory effects of cholecalciferol supplementation on total adiponectin concentrations [27], it has been shown in obese children that VDD is associated with lower expression of adiponectin isoforms, particularly HMW-A, while 1 year supplementation with cholecalciferol was shown to upregulate HMW-A expression independent of changes in BMI [28]. Pediatric studies also found that the increase in HMW-A levels was correlated with improvements in insulin sensitivity [29,30]. Finally, cholecalciferol supplementation in children was shown to decrease the value of the leptin-to-adiponectin ratio (L/A) [31], a marker of metabolic disease [32,33], which is better correlated with insulin resistance than the levels of each single peptide [34,35]. 

Based on the evidence that cholecalciferol may improve insulin sensitivity, we sought to explore the effect of 25(OH)Vit D on glucose homoeostasis in non-diabetic obese subjects with VDD through analysis of indirect mediators of insulin sensitivity, such as adiponectin oligomers. We therefore undertook a placebo-controlled investigation to explore if the administration of a large dose of cholecalciferol in severely obese individuals could acutely modify the profile of adiponectin oligomers measured by western immunoblot and ELISA, in relation to anthropometric measures, indices of insulin resistance, and the leptin-to-(multimeric) adiponectin ratio.

## 2. Materials and Methods

### 2.1. Subjects

The study enrolled 24 obese patients (13 males/11 females; age 37.5 ± 1.9 years; BMI 41.3 ± 0.8 kg/m^2^) who were referred to our institution for work-up and rehabilitation of obesity and its comorbidities. All patients were enrolled between July 2014 and February 2015 after biochemical demonstration of VDD status and were distributed during the three subsequent seasons. Written consent was obtained from all patients after full explanation of the purpose and nature of the study. The investigation was approved by the local ethical committee (Comitato Etico, Istituto Auxologico Italiano, Milano, Italy, 18F101_2011), and was developed in accordance with the 3rd edition of the Guidelines on the Practice of Ethical Committees in Medical Research.

Patients were otherwise healthy, and exclusion criteria included T1DM or T2DM, autoimmune or inflammatory diseases, kidney or cardiac disorders, chronic steroid treatment, any therapy capable of influencing calcium metabolism, vitamin D treatment, and comorbidities affecting 25(OH)Vit D metabolism, and menopause. Patients had not been prescribed any pharmacological therapies, diet therapy, dietary supplements, or anti-obesity compounds for at least 3 months prior to entering the study. Obese subjects were studied at baseline upon admission and following a four-week inpatient metabolic rehabilitation consisting of individualized caloric restriction equivalent to 75% of basal resting energy expenditure measured by indirect calorimetry, physical exercise comprising three sessions per week of aerobic activity, supported by a nutrition and lifestyle action program consisting of three 1-h classes per week dedicated to an educational approach on dietary behavior, nutrition knowledge, and motor activity. A standard inpatient hypocaloric diet consisting of 30% lipids, 50% carbohydrates, and 20% proteins was administered during the study, and the average caloric deficit was 488 ± 79 kcal/day for the VD group and 485 ± 83 kcal/day for the PL group. Neither supplements nor anti-obesity therapies were used during the study period.

### 2.2. Study Design

On admission, patients underwent routine analysis and assessment of VDD considered as 25(OH) Vit D levels <20 ng/mL [36]. Subsequently, patients were randomly allocated to a single blind, placebo–controlled study. The starting study population included 26 subjects with VDD, two of whom dropped out during the study. The remaining 24 patients were divided between the vitamin D group (VD group; 6 males/6 females; age 38 ± 2.4 years; BMI 42.7 ± 1.3 kg/m^2^) and the placebo group (PL group; 7 males/5 females; age 37 ± 3 years; BMI 39.8 ± 0.9 kg/m^2^). An effort was made to include patients with comparable age, gender, and BMI in the two groups. Patients of the VD group underwent administration of an oral dose of commercially available oily solution containing 600,000 IU of cholecalciferol. Patients of the PL group were administered an equivalent volume of certified cholecalciferol-free olive oil. Following administration, fasting blood samples were collected for analysis at baseline and after 3, 7, 14, and 28 days post-administration in each group. 

### 2.3. Body Measurements

All subjects underwent body measurement wearing light underwear in fasting conditions after voiding. Weight and height were measured to the nearest 0.1 kg and 0.1 cm respectively. BMI was expressed as body mass (kg)/height (m)^2^. Obesity was defined for any BMI over 30 kg/m^2^. Waist circumference was measured midway between the lowest rib and the top of the iliac crest after gentle expiration; hip circumference was measured as the greatest circumference around the nates. Anthropometric data were expressed as the mean of two measurements and were assessed at study entry and at study end. At baseline, all subjects underwent dual-energy X-ray absorptiometry (GE-Lunar, Madison, WI, USA) for measurement of lean and fat body mass the morning after an overnight fasting and after voiding.

### 2.4. Western Immunoblot (WIB) of Multimeric Adiponectin

Blood samples were drawn in fasting conditions, then they were centrifuged, separated, aliquoted, and kept at −80 °C until assays in single batches. As previously published [28], for WIB all serum samples were size-fractionated on 10% Sodium Dodecyl Sulphate PolyAcrylamide Gel Electrophoresis (SDS-PAGE) under non-reducing (NR) conditions and electro-transferred to immuno-blot polyvinylidene difluoride (PVDF) membranes (BioRad, Hercules, CA, USA). Membranes were incubated with monoclonal anti-adiponectin (Adipogen, Incheon, Korea) and detected with the appropriate horseradish peroxidase-conjugated secondary antibody (Chemicon Millipore, Temecula, CA, USA). Immunoreactive proteins were detected using enhanced chemiluminescence (Pierce Biotechnology, Rockford, IL, USA) with image capture performed using a CCD-camera linked to ChemiTouch (BioRad). The results were quantified using Image Lab software (BioRad). Values are presented as arbitrary units (AU) normalized to total protein determined by Ponceau S staining (Sigma Aldrich, St. Louis, MO, USA). 

### 2.5. Adiponectin Immunoassay

Serum total adiponectin and adiponectin multimeric forms were determined as previously published [20], using the Adiponectin (Multimeric) Enzyme Immunoassay (ALPCO Diagnostics, Salem, NH, USA), according to the manufacturer’s instructions. Assay sensitivity is 0.019 ng/mL, the limit of detection performed is 0.0196 ng/mL; as reported by the manufacturer, overall intra- and inter-assay coefficient of variations (CV) for total, HMW, and MMW + HMW are 5.4–5.0%, 5.0–5.7%, and 5.0–6.0%, respectively. Total adiponectin and HMW-A were measured directly from the assay plates, whereas MMW-A and LMW-A were calculated; MMW-A was obtained by subtracting the concentration of HMW-A from the combined concentration of MMW-A and HMW-A (measured directly), and LMW-A was obtained by subtracting the combined concentration of MMW-A and HMW-A (measured directly) from the total concentration of adiponectin. In parallel to sample preparation, duplicate P1 (protease one of the kit) digestions were also prepared for WIB under non-reduced conditions according to the manufacturer’s instructions. 

### 2.6. Biochemistry

Laboratory data were obtained in a central laboratory. Blood glucose and HbA_1c_ were measured by enzymatic methods (Roche Molecular Biochemicals, Mannheim, Germany). HbA_1c_ values of 6.5% were considered diagnostic for T2DM. Insulin (CVs, 0.8–1.5% and 2.4–4.9%) was measured using a Cobas Integra 800 Autoanalyzer (Roche Diagnostics, Indianapolis, IN, USA). In both groups, insulin resistance was calculated by the homeostatic model of insulin resistance (HOMA-IR) index as insulin (mU/L) × (glucose (mmol/L)/22.5). 25(OH)Vit D levels were determined by an Agilent HP1100 series HPLC system equipped with a Variable Wavelength Detector; the detector wavelength was set at 265 nm. 25(OH)-Vit D was determined using an analysis kit from Chromsystems (Chromsystems Instruments & Chemicals GmbH, Gräfelfing, Germany). Calibration was performed using a lyophilized serum calibrator NIST traceable of known concentration. Low and high concentration lyophilized sera samples were used as quality controls. Samples, calibrators, and quality controls were prepared according to the manufacturer’s instructions. Assay sensitivity was 1.4 µg/L and the inter- and intra-assay coefficients of variations were 0.9–3.0% and 2.3–3.3%, respectively. Serum leptin levels were measured using a Leptin Human ELISA kit (Mediagnost, Ruetlingen, Germany) with a sensitivity of 0.2 ng/mL, and inter- and intra-assay coefficients of variability below 10%, as reported by the manufacturer.

### 2.7. Statistics

Data are expressed as the mean ± SEM. Statistical analyses were carried out by GraphPad Prism 5 (GraphPad Software, La Jolla, CA, USA) using unpaired and paired two-tailed Student’s *t*-test to compare baseline characteristics of the two groups and within the obese population at the different time-points. One-way ANOVA for repeated measures was used to assess within-group modifications of measured variables during the study. Two-way ANOVA was performed to analyze the effect of time, treatment, and time × treatment. Assessments of differences between groups were additionally assessed by the χ^2^ test. Linear regression per Pearson’s analysis was performed to determine correlation coefficients between different parameters. *p* < 0.05 was considered as statistically significant. Percent ∆ value was calculated as the final value − baseline value/baseline value × 100.

## 3. Results

### 3.1. Metabolic Effects of Cholecalciferol Supplementation

The characteristics of the 24 participants enrolled in the two study groups are shown in Table 1. 

**Figure 1:**
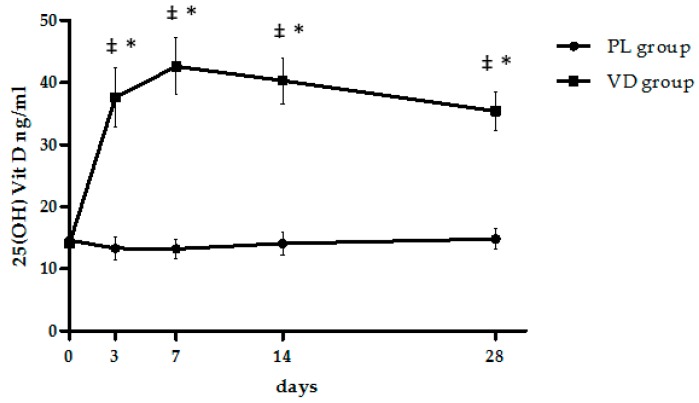
Effect of cholecalciferol or placebo administration on serum 25(OH)Vit D levels. Data are presented as mean ± SEM. For significance: * *p* < 0.001 vs. baseline within group, as calculated by repeated measures one-way ANOVA; ^‡^ *p* < 0.0001 between groups by unpaired *t*-test. Two-way ANOVA was also performed to test the effect of time, treatment, and time × treatment interaction, and results are summarized in the text and Table 1.

We found no difference in anthropometric variables between the VD group and the PL group at baseline, including BMI and body weight. A slight but non-significant difference in BMI (*p* = 0.08) and percent fat mass (*p* = 0.07) occurred between groups. Baseline levels of 25(OH)Vit D were similar across groups, and all subjects were 25(OH)Vit D deficient, considered as 25(OH)Vit D <20 ng/mL. Metabolic analysis showed baseline glucose levels in the diabetic range in one patient in the VD group. Baseline values of leptin and total adiponectin, as well as their ratio, were comparable between groups. Figure 1 shows changes in 25(OH)Vit D levels following cholecalciferol or placebo administration in the study populations. 

Figure 1 shows the changes in serum 25(OH)Vit D levels induced by cholecalciferol or placebo supplementation in the studied populations. A significant change in 25(OH)Vit D levels occurred throughout the entire observation period only in the VD group, and analysis by repeated measures ANOVA showed that this change was significant (*p* < 0.001). In particular, a sharp and significant increase in 25(OH)Vit D levels was observed as soon as after 3 days following administration and persisted at significantly higher levels than baseline throughout the study period. By contrast, there was no modification in 25(OH)Vit D levels in the PL group. The interaction between cholecalciferol treatment and time was significant (*p* < 0.0001). At the end of the treatment period, obese patients treated with cholecalciferol lost 6 ± 0.7% of their body weight (Table 1), which did not statistically differ from the 5.5 ± 0.5% weight loss observed in the PL group. Overall changes of immunoassayable total adiponectin levels were negligible, whereas leptin levels significantly decreased in both groups. Hence, a significant reduction was also documented in the L/A ratio in both groups.

### 3.2. Analysis of Multimeric Adiponectin

**Figure 2:**
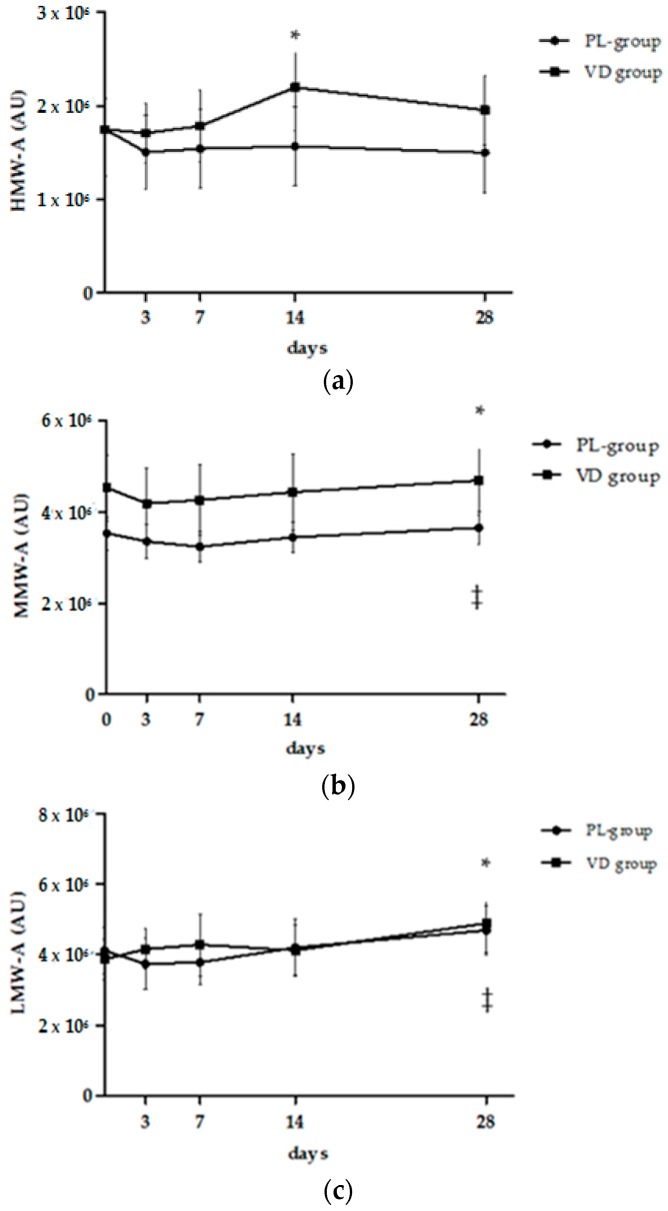
Effect of cholecalciferol or placebo administration on serum adiponectin oligomer expression levels measured by western immunoblot. Data are presented as mean ± SEM. Values are presented as arbitrary units (AU) normalized to total protein determined by Ponceau S staining. (**a**) High molecular weight adiponectin (HMW-A): for significance in the VD group: * *p* < 0.05 vs. baseline, 3 and 7 days after treatment (repeated measures one-way ANOVA), no difference between groups by unpaired *t*-test; (**b**) medium molecular weight adiponectin (MMW-A): for significance: * *p* < 0.05 vs. 3 days in the VD group; ^‡^ *p* < 0.05 vs. 7 days in PL group (repeated measures one-way ANOVA), no difference between groups by unpaired *t*-test; (**c**) low molecular weight adiponectin (LMW-A): for significance: * *p* < 0.05 vs. 3, 7, and 14 days in the VD group; ^‡^ *p* < 0.05 vs. 3, 7 days in the PL group (repeated measures one-way ANOVA), no difference between groups by unpaired *t*-test. Two-way ANOVA was also performed to test the effect of time, treatment, and time × treatment interaction, and results are summarized in the text.

**Figure 3:**
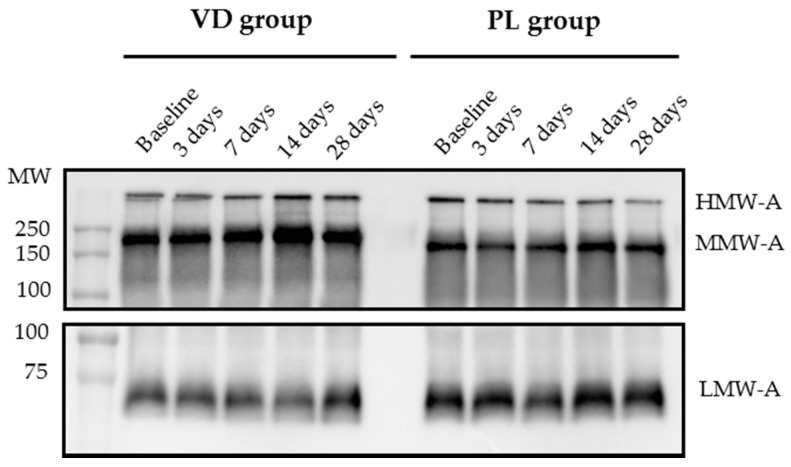
Representative immunoblots of high (HMW-A), medium, (MMW-A), and low molecular weight adiponectin (LMW-A) changes obtained at baseline and after 3, 7, 14, and 28 days following cholecalciferol or placebo administration in obese subjects. Representative western immunoblot (WIB) analyses under non-reduced conditions are displayed. HMW-A and MMW-A were analyzed following P1 digestion, while LMW-A was analyzed directly (for description see text). MW = molecular weight.

Sera obtained from obese subjects undergoing the two treatment arms were processed by WIB to assess whether cholecalciferol treatment influenced adiponectin multimerization as compared to placebo. Despite a visual difference in baseline MMW-A values, analysis of multimeric adiponectin at study entry showed no difference between groups in the expression levels of HMW-A, MMW-A, and LMW-A. During the study, individual HMW-A profiles displayed a progressive increase in 71% of patients treated with cholecalciferol compared to 36% of those of the PL group (χ^2^ = 24.6, *p* < 0.0001). A cumulative increase in HMW-A expression, depicted in Figure 2 and Figure 3, was only evident for the VD group but reached statistical significance 14 days following cholecalciferol administration (*p* < 0.05 vs. baseline, 3 and 7 days). Inversely, we failed to document any significant interaction between time and treatment, probably due to the low sample number. An incremental trend was also observed for MMW-A and LMW-A, but this occurred in both groups, regardless of cholecalciferol treatment. Specifically, a significant change in MMW-A and LMW-A expression was achieved at 28-day when compared to the day 3 time-point (*p* < 0.05), thus likely suggesting a diet-related effect.

Based on WIB analysis, we then sought to corroborate results obtained at baseline and after 14 and 28 days by multimeric adiponectin immunoassays. As shown in Table 2, we failed to document significant increments in HMW-A in the VD group, while in the PL group there was a slight decrease of HMW-A at 28-day. By contrast, MMW-A and LMW-A did not appear to consistently change in either group during the experimental period. For all three adiponectin oligomers, we did not document significant effects of cholecalciferol administration, time of treatment, and their interaction by two-way ANOVA. Overall, one-way ANOVA for repeated measures also showed no significant difference. When the leptin/HMW-A ratio was used as a surrogate measure of the bioactive adiponectin multimerization, a decrease was only documented in the VD group (Table 1).

No correlation was observed when we analyzed the effect of vitamin D_3_ treatment in relation to adiponectin isoforms, body weight, and measures of insulin resistance, when assessed as both absolute values and incremental changes. 

## 4. Discussion

Recent research has demonstrated that 25(OH)Vit D modulates the secretion of adipokines such as leptin and adiponectin [28,37]. In particular, the most bioactive form of adiponectin, HMW-A, was found to be down-regulated in obese children with VDD, and to increase following cholecalciferol supplementation [28]. Current results suggest that oral administration of a high dose of cholecalciferol to obese adults with VDD did not significantly impact total adiponectin concentrations but promoted a selective increase of the HMW-A oligomer, which was independent of initial body weight, insulin resistance, and amount of body weight lost on diet. Because HMW-A is a putative modulator of insulin homeostasis, and its changes were specific to cholecalciferol treatment, it seems conceivable that cholecalciferol may intervene on molecular intermediates of glucose homeostasis and influence insulin sensitivity.

Obesity is a global health problem and an important risk factor for diabetes, cancer, heart disease, and hypertension. Obesity and the metabolic syndrome have a high impact on the current epidemic of VDD [38]. Owing to its pleiotropic actions, supplementation with vitamin D_3_ and its congeners is hypothesized to act beneficially on several markers of chronic diseases [39]. It has been shown that serum 25(OH)Vit D concentrations are inversely correlated with body mass index (BMI), fat mass, or percentage of body fat, as well as waist circumference [2]. It is not clear whether this association is due to increased storage in the adipose tissue, sedentary lifestyle, low sunlight exposure, true vitamin D deficiency, genetic changes in vitamin D metabolism, or unknown factors [38]. 

Interventional studies investigating 25(OH)Vit D action on weight gain [40], glucose abnormalities [41], and lipid profiles [42] failed to provide reproducible results. However, problems remain with the daily amount of vitamin D_3_ supplementation, which was in these studies lower than the vitamin D intake considered to be appropriate for adults [43,44]. Current evidence collected from randomized trials does not support an effect for short-term vitamin D supplementation on glucose control in a heterogeneous population with type 2 diabetes, whereas a favorable effect of vitamin D on fasting glucose was seen in patients with poorly controlled diabetes [9]. In overweight and obese subjects, high dose vitamin D_3_ supplementation was also reported to amplify the beneficial effect of weight loss on traditional and non-traditional cardiovascular disease risk markers, independent of changes in body weight, fat mass, and abdominal fat mass [45]. In agreement with previous findings [46,47], our study observed that administration of a high cholecalciferol dose in VDD obese subjects caused a prompt and sustained increase of 25(OH)Vit D levels for the 28-day study period. The main finding of our study was the statistically significant incremental modification of HMW-A expression detected by western immunoblot after cholecalciferol administration but not after placebo. This increment appeared slightly delayed as compared to the peak of 25(OH)Vit D levels in the circulation, thus the peak 25(OH)Vit D levels occurred after 7 days while HMW adiponectin peaked after 14 days post-administration, thus suggesting a possible genomic effect. By two-way ANOVA, however, there was no time × treatment interaction, likely due to the small sample. We found a similar incremental effect exerted by cholecalciferol and placebo on MMW-A and LMW-A profiles, which was likely consequent to the diet-induced weight loss. While it should be noted that the placebo group tended to have slightly milder baseline measures of severe obesity and metabolic dysfunction compared to the vitamin D group, these differences were not statistically significant and, thus, we hypothesize that this divergence played a negligible effect on the main findings of this study. In support of our findings obtained by WIB on cholecalciferol and placebo, previous studies showed that supplementation with vitamin D_3_ at therapeutic doses did not impact total adiponectin concentrations [27], but it selectively increased HMW-A levels in obese adolescents treated for 1 year with cholecalciferol [28]. While our study and other studies failed to record associations between short-term increases of HMW-A and improvements in insulin sensitivity [20,21], long-term studies documented a significant correlation between modifications of HMW-A and insulin sensitivity [29,30,48]. Based on our study and long-term clinical data, we hypothesize an insulin-sensitizing effect of vitamin D supplementation through HWM-A. 

At odds with results obtained by WIB, our investigation on multimeric adiponectin quantitated by ELISA failed to capture modifications of HWM-A related to cholecalciferol administration, nor did it detect the increases of MMW-A and LMW-A identified by WIB in both groups. There are no comparative data in the literature that fully address this issue, and it cannot be excluded that it may depend on the performance of the methodologies herein used. We, thus, speculate that this discrepancy may reflect intrinsic differences between qualitative/semiquantitative (western immunoblot) and quantitative (ELISA) analyses. This incongruence has been previously noted in a study by Polack and colleagues, where an increase in all three types of adiponectin multimeric complexes was identified by western immunoblot but it was not accompanied by significant changes in total plasma adiponectin levels measured by ELISA [21]. Finally, such a discrepancy could also reflect different binding capacities of the different monoclonal antibodies used by the two methods.

From a mechanistic viewpoint, adipose tissue is indeed a major site of vitamin D storage, and both the VDR and vitamin D metabolizing enzymes are expressed in adipocytes. Partly contradictory results exist on the adipogenic effect of 1,25(OH)_2_Vit D [37], which was shown to inhibit adipogenesis in 3T3-L1 mouse preadipocyte cell lines [49], while promoting adipogenesis and increasing adiponectin expression in primary mouse and human subcutaneous preadipocytes [50]. Similarly, treatment with 1,25(OH)_2_Vit D in 3T3-L1 cells was found to stimulate the synthesis of adiponectin and its multimeric forms [27]. Moreover, a key regulator of folding and assembly of adiponectin, the endoplasmatic reticulum (ER) chaperon DsbA-L, was found to be upregulated by 1,25(OH)_2_Vit D, which suggests that vitamin D regulation of adiponectin may involve post-transcriptional mechanism/s [28]. Alternatively, no effect of 1,25(OH)_2_Vit D on adiponectin expression was found in human adipocyte cultures [51], suggesting that the effect of vitamin D on adiponectin secretion is on open field and warrants further studies.

Our findings also indicate that, in the short term, vitamin D_3_ supplementation had no additional effect on the magnitude of weight loss compared to placebo, which agrees with previous evidence that vitamin D_3_ supplementation does not promote improvements in the degree of obesity [52]. On the other hand, we also observed that weight loss obtained without Vitamin D_3_ supplementation did not modify circulating 25(OH)Vit D levels, which agrees with the inference that loss of adipose tissue does not promote the release of 25(OH)Vit D release from fat into the bloodstream [53]. Although vitamin D_3_ supplementation does not act synergistically on adiponectin and leptin levels [54], we found a reduction of the leptin/adiponectin ratio in both groups, while a reduction of the leptin/HMW-A ratio was only obtained in the VD group. Based on the predictive role of these measures on metabolic health [32,33,34,35] and response to anti-diabetic therapy [55], such a result may add strength to the metabolic role of vitamin D_3_ administration, independent of changes in body weight. This suggestion is substantiated in studies on obese adolescents, where a 6-month cholecalciferol supplementation (4000 IU/day) significantly decreased the leptin/adiponectin ratio in the absence of changes in BMI or waist circumference [31]. 

As limitations of our study, we acknowledge that the small sample size may hamper the clinical significance of our observations. However, the randomized placebo-controlled design of the study is a potential point of strength in addition to the tight monitoring of study effects during the inpatient dieting program. As all participants received a weight loss intervention leading to similar outcomes between groups, we could discriminate independent effects of vitamin D_3_ supplementation in our experimental conditions. Indeed, the duration of the study and the obese sample does not allow for insights to be gained on the long-term effects vitamin D_3_ supplementation, or extend our findings to lean populations and different clinical settings. Finally, our study cannot provide insights on translational and post-translational mechanisms involved in modifications of adiponectin oligomers. 

## 5. Conclusions

In conclusion, we found that an acute dose of cholecalciferol in obese VDD subjects promoted changes of HMW-A independent of changes in body weight and insulin resistance. We observed a decrease in the ratio between leptin and adiponectin or HMW-A. These results could not be confirmed by immunoassay procedures. Further studies are needed to clarify the mechanisms underlying these effects.

## Figures and Tables

**Table 1:** Summary of anthropometric and metabolic data in the two populations obtained at baseline and at the end of the study.

	Vitamin D Group (*n* = 12)			Placebo Group (*n* = 12)			*p* Value		
	Baseline	At the end of the study	∆ (%)	Baseline	At the end of the study	∆ (%)	Time	Treatment	Time × Treatment
Male/Female	6/6	-	-	7/5	-	-	-	-	-
Age (years)	38 ± 2.4	-	-	37 ± 3.0	-	-	-	-	-
BMI (kg/m^2^)	42.7 ± 1.3	40.1 ± 1.2 **	−6.0 ± 0.7	39.8 ± 0.9	37.6 ± 0.7 **	−5.5 ± 0.5	<0.0001	0.07	0.41
Body weight (kg)	115.9 ± 4.4	109.2 ± 4.9 **	−5.8 ± 0.4	112.7 ± 4.7	106.4 ± 4.3 **	−5.5 ± 0.5	<0.0001	0.64	0.65
Fat mass (%)	44.8 ± 1.6	-	-	40.4 ± 1.6	-	-	-	-	-
Free fat mass (kg)	63.4 ± 2.9	-	-	66.7 ± 3.3	-	-	-	-	-
Waist (cm)	126.2 ± 3.5	-	-	119.0 ± 4.0	-	-	-	-	-
Glucose (mg/dL)	97.0 ± 7.1	90.2 ± 3.1	−7.1 ± 4.1	88.5 ± 3.2	83.0 ± 1.6 *	−5.5 ± 2.1	0.07	0.45	0.10
Insulin (mU/L)	13.9 ± 2.5	14.5 ± 1.7	5.9 ± 12.7	17.5 ± 2.2	14.1 ± 1.3	−18.5 ± 8.1	0.06	0.17	0.30
HbA_1c_ (%)	5.5 ± 0.1	5.7 ± 0.3	0.7 ± 3.2	5.6 ± 0.6	5.3 ± 0.2 *	−4.7 ± 1.3	0.21	0.53	0.07
HOMA−IR	3.9 ± 0.6	3.3 ± 0.4	−3.2 ± 11.3	3.7 ± 0.5	3.0 ± 0.2	−9.7 ± 8.3	0.11	0.67	0.99
25(OH)Vit D (ng/mL)	14.2 ± 1.9	35.0 ± 3.2 ^#,^**	191.0 ± 39.6	14.5 ± 1.9	14.8 ± 1.6	7.2 ± 6.3	<0.0001	<0.0001	<0.0001
Total adiponectin (µg/mL)	3.6 ± 0.5	3.5 ± 0.4	0.2 ± 5.9	4.0 ± 1.1	3.9 ± 1.0	1.9 ± 9.1	0.39	0.75	0.89
Leptin (ng/mL)	44.1 ± 7.2	31.6 ± 5.9 *	−27.3 ± 5.4	41.1 ± 4.6	9.8 ± 4.1 **	−29.9 ± 4.0	<0.0001	0.75	0.72
Leptin/adiponectin	15.9 ± 4.3	10.5 ± 2.7 *	−23.6 ± 5.4	14.8 ± 2.5	10.3 ± 1.6	−21.2 ± 12.9	<0.01	0.86	0.73
Leptin/HMW−A	49.1 ± 12.0	24.7 ± 4.2 *	−26.8 ± 10.6	37.4 ± 7.4	35.5 ± 9.9	−6.9 ± 12.8	<0.05	0.96	<0.05

Data are presented as mean ± SEM. *p*-values refer to the effect of time, treatment, and time × treatment assessed by two-way ANOVA. For significance: a paired *t*-test was performed in each group between baseline and study-end assessment (** *p* < 0.001, * *p* < 0.05); an unpaired *t*-test was performed between the two groups at baseline and at the end of the study (^#^ *p* < 0.01). For abbreviations: BMI, Body Mass Index; HbA_1c_, Glycated Haemoglobin; HOMA-IR, Homeostatic Model of Insulin resistance; 25(OH)Vit D, 25-hydroxycholecalciferol; HMW-A, High Molecular Weight Adiponectin; ∆ (%), percent delta value (listed as mean ± SEM).

**Table 2:** Biochemical evaluation of multimeric adiponectin by ELISA.

	Vitamin D Group (*n* = 12)			Placebo Group (*n* = 12)			*p* Value		
	Baseline	14 Days	28 Days	Baseline	14 Days	28 Days	Time	Treatment	Time × Treatment
**Total Adiponectin (µg/mL)**	3.6 ± 0.5	3.7 ± 0.4	3.5 ± 0.4	4.0 ± 1.1	3.9 ± 0.9	3.8 ± 1.0	0.94	0.51	0.99
**HMW-A (µg/mL)**	1.6 ± 0.4	1.6 ± 0.2	1.5 ± 0.2	2.1 ± 0.8	1.9 ± 0.5	1.8 ± 0.7	0.92	0.38	0.97
**MMW-A (µg/mL)**	0.7 ± 0.1	0.6 ± 0.1	0.7 ± 0.1	0.5 ± 0.2	0.4 ± 0.2	0.8 ± 0.2	0.63	0.93	0.87
**LMW-A (µg/mL)**	1.5 ± 0.2	1.5 ± 0.2	1.4 ± 0.2	1.5 ± 0.3	1.8 ± 0.4	1.5 ± 0.4	0.64	0.51	0.74

Data are represented as mean ± SEM. *p*-values refer to the effect of time, treatment, time × treatment assessed by two-way ANOVA. No significant difference between groups was assessed at each time-point by unpaired *t*-test. For abbreviations: HMW-A, High Molecular Weight Adiponectin; MMW-A, Medium Molecular Weight Adiponectin; LMW-A Low Molecular Weight Adiponectin.
